# An Account of the Means Successfully Employed in Preserving the Health of the Seamen, on Board His Majesty's Ship Astrea; on the Jamaica Station, in the Years 1787, 1788, 1789, and Part of 1790

**Published:** 1799-03

**Authors:** Stewart Henderson

**Affiliations:** formerly a Surgeon in the Royal Navy, now of the Army Hospital-Staff


*** *The following interesting communication is deemed worthy of a place in our first number. It unfortunately did not reach us till the part of the Journal appropriated to such Original Communications had been printed off.*

### *An Account of the Means successfully employed in Preserving the Health of the Seamen, on board his Majesty's Ship Astrea; on the Jamaica Station, in the Years* 1787, 1788, 1789, *and Part of* 1790*:—By* STEWART HENDERSON, *formerly a Surgeon in the Royal Navy, now of the Army Hospital-Staff.*

WHEN we look into the political and medical history of this war, we are at a loss to say, which of the two great enemies of mankind, French principles, or the pestilential disease, has been most destructive. While it becomes the province of the statesman to enact salutary laws, to prevent the baneful influence of principles subversive of all social order, it is no less the duty of the guardians and superintendants of health, to recommend and employ such precautions and means, as prudence and experience have suggested, to prevent the introduction, and check the progress of a formidable enemy—pestilential disease; which commits such ravages on the human race, and seems even yet to bid defiance to the medical art.

The prevention of disease often depends on circumstances, which to some appear trifling, and, therefore, are but little attended to: they are, however, found by experience to be of eminent utility and importance. This, I trust, will be shewn, in the following faithful account of the Astrea; being an extract from my Medical Register, with practical remarks on the diseases; which I intended to have offered to the public, on my arrival from the West Indies, in April 1790, had leisure permitted.

Previous to giving an accouut of the means employed on board the Astrea, I think it proper first to observe, that the great and celebrated circumnavigator, Captain COOKE, proved to the world the possibility of carrying a ship's crew through a variety of climates, for the space of near four years, without losing one man by disease; a circumstance which added more to his fame, and is supposed to have given a more useful lesson to maritime nations, than all the discoveries he ever made. He has shewn, that health may be preserved, on the most distant expeditions, undertaken either for the purposes of war or commerce, and by means no less simple than efficacious. It is true, he possessed advantages, which few ships of war, on any service, can expect to have have; a large roomy ſhip, to contain a ſmall number of choſen men, all volunteers, provided with every neceſſary, and cloathing adapted to different climates. Articles of diet, and preventives, which other ſhips are not ſupplied with, and the proviſions of a ſuperior quality to what is generally allowed; above all, the ſhip almoſt conſtantly at ſea, and not obliged to put into unhealthy harbours.

Other judicious and ſkilful commanders, who have ſince adopted the means he purſued, have met with equal ſucceſs, though under leſs favourable circumſtances; this, however, does not diminiſh the juſt praiſe of that illuſtrious navigator, who well merited the honorary medal beſtowed upon him by the Royal Society, and preſented by the preſident, Sir JOHN PRINGLE.

His Majeſty's ſhip Aſtrea, of 32 guns, and 200 men (peace complement), commanded by Captain PETER RAINIER (now Vice-admiral, commander in chief of his Majeſty's ſhips in India), was fitted out at Woolwich, in October 1786, and ſailed for Jamaica the 6th of January, 1787. I did not join her until a few days before ſhe ſailed, ſo that I had it not in my power to ſelect the medicines moſt uſeful for the climate. My predeceſſor had previouſly ordered the medicine-cheſt from the Apothecaries' Company, perhaps without knowing her intended ſtation. I found, however, that the men were judiciouſly ſelected, many of them having lately returned from the Weſt Indies. Upon inſpecting the whole of the crew, I had occaſion to requeſt ten men might be ſent to Haſlar Hoſpital, as being unfit ſubjects for a hot climate, and, indeed, for the ſervice; being affected chiefly with ruptures and old ulcers.

We were not allowed any preventive articles of diet; no ſour-crout, eſſence of ſpruce, or malt, orange or lemon-juice, potatoes, ſugar, or wine for the ſick; which neceſſary articles have been ſince liberally ſupplied to his Majeſty's ſhips, in conſequence of the able repreſentations of Dr. TROTTER, phyſician to the fleet, and other medical gentlemen in the navy; there is now, likewiſe, a better allowance of vegetables when in port; which, as appears from the medical hiſtory of the fleet, has had a very ſalutary effect.

As we could not call in theſe auxiliaries, it behoved us to redouble our exertions, and to beſtow the greateſt attention on thoſe in our power, and which we knew from experience to be moſt uſeful. I judged that the health of the men, and exemption from the diſeaſes of the climate, would, in a great degree, depend on cleanlineſs, ventilation, keeping the deck below free from moiſture, cold-bathing, preventing irregularities, and expoſure to marſh-effluvia; conſtant employment for the body and mind; but yet to avoid working in the ſun before the ſea-breeze ſet in; and to provide, when the ſervice would admit, the liberal uſe of vegetables and other antiſeptics.

Such

Such were the means I propofed for the prevention of difeafe; and from Captain Rainier's character, as a humane and experienced officer, and his long acquaintance with hot climates, I had every reafon to expect, that whatever would tend to a falutary effect, would meet with his approbation and fupport.

I foon found that he well underftood that important part of an officer's duty; and it is from his judicious orders, regulations, and attention on the part of the officers, in feeing them well executed, the means employed for preferving and reftoring health were fo fuccefsful, that not one of the fhip's company died on board, and only four were at the hofpital from difeafe during the three years the fhip remained on the Jamaica ftation: a great part of that period we lay in a very unhealthy harbour (Kingfton); a fingular circumftance, contrafted with what has fince happened in that part of the world.

On the paffage, we ftopped fourteen days at Ferrol and Corunna, where we were fupplied with frefh beef and vegetables; and likewife at Madeira, where we remained a week. The men, as ufual, were put into divifions or fquads, under certain officers, who infpected their cloathing, to fee that they kept themfelves clean and orderly, particularly on Sundays, when they were muftered by the commanding officer. During the whole time of our lying on the ftation, cold-bathing was almoft daily ufed. When in harbour, a fail was put over-board, funk to a certain depth, and fufpended from the yard-arm, to prevent danger from the fharks, or any other accident to fuch as might not have learnt to fwim. When the lower deck was wafhed, fires were kept in Brodie's ftoves, until it was completely dried, and well fumigated by the ufual mode, as well as in rainy weather, or when there was much moifture in the atmofphere. A fire was likewife put into the well, after being fwabbed dry, which was more effectual in deftroying the noxious air, than the cuftom of letting in water by the pumps. To prevent thofe irregularities on fhore, which too often difgrace the character of Britifh feamen and foldiers, and expofe them to the exciting caufes of difeafe, Captain Rainier very prudently refufed any leave of that kind to men he could not depend upon. Indeed, after a few months abftinence, they did not feem folicitous to obtain it; knowing, they had no money to purchafe their favourite, but baneful liquor, or power to fell their neceffaries, with impunity. I believe the Aftrea was the only fhip in the navy, which had no occafion for what is called a liberty-book, while the crew feemed perfectly fatisfied and contented without that permiffion; a proof, I think, of their being under excellent difcipline. It is true, they were allowed on Sundays, when in port, to vifit their acquaintance in the different fhips in the fquadron, and on thofe occafions a moft complete fcene of drunkennefs was ufually exhibited; fcarcely a man but was obliged to be hoifted on board, and in a fituation that required no little management management to prevent fatal confequences. On thofe days I thought it neceffary to be on board, and to give directions that they fhould be placed properly in their hammocks. This neceffary indulgence, however, was fometimes productive of ficknefs, but not of that tendency which would have happened, had the fame inebriety been committed on fhore; and we confidered, that conftant confinement might have had a worfe effect, than the irregularities they were guilty of. When not employed on duty, amufements and diverfions were promoted and encouraged by the officers. Neither was there any reftriction to women coming on board; which did away one principal motive for their wifhing to go on fhore.

To guard as much as poffible againft the bad effect of marfh effluvia, the fhip was always anchored as far from the fhore, as could in fafety be admitted; and in the different iflands we vifited, St. Domingo, Porto-Rico, Curaffoa, Cuba, &c. care was taken to procure frefh beef, and plenty of fifh, by hauling the *feine*. This laft was never neglected, and it proved to be a very nutritious and proper food in thofe countries. They were likewife fupplied with vegetables, oranges, and limes, which grow in many parts fpontaneoufly; thefe were procured in abundance, and when it became abfolutely neceffary to employ the fhip's company in wooding, (which fhould never be done, if poffible, in hot climates,) the party were injoined to be on board before dark, by which they efcaped the bad effects of the night air. This fervice has frequently occafioned the lofs of many feamen. The watering duty, another fervice very deftructive to the health of feamen, was performed by blacks, who manned the long-boat, and whofe conftitutions were congenial to the climate.

Thefe were the principal means we employed for preferving the health of our men; the confequence was, as I have already related, that not one of the crew died on board; a fact, which may be feen by the fhip's books in the Navy-office; though ficknefs will always prevail more or lefs, on board of fhips, from caufes which no human exertions can prevent. I am perfuaded alfo, that in time of war, from the nature of the fervice carrying on, the regulations before ftated, and the obfervance of them, cannot be fo ftrictly adhered to; yet I am convinced, from what I obferved during nearly the five years I was employed in the Weft-Indies laft war, that a great deal of ficknefs might have been prevented, and many valuable lives preferved, had fuch falutary means been better underftood, and attended to.

This, in my opinion, points out the neceffity of a code of falutary regulations being eftablifhed, for the guidance of commanding officers of his majefty's fhips, who may not have had an opportunity of becoming acquainted with the means of prevention, and are liable to err, from a miftaken idea of the caufes of difeafe. Should any general ficknefs prevail, an enquiry might be inftituted, to learn whether it proceeded from neglect, or unavoidable caufes.

GENERAL

GENERAL STATE OF THE WEATHER.

As the weather is juftly fuppofed to have a confiderable effect in producing difeafe, I think it neceffary to give fome account of it, during the period of three years we remained on the Jamaica ftation.

I have already obferved, that the Aftrea failed from England for Jamaica in the beginning of the year 1787. Except three or four flight venereal cafes, we had no fick on board, having fent to the Hofpital thofe whom we deemed improper fubjects for the fervice. In the channel we experienced a great deal of cold, boifterous weather, before we reached Corunna; during which time four men were feized with pulmonary complaints, and one with acute rheumatifm. After leaving the coaft of Spain, we arrived at that delightful and falubrious ifland, Madeira. As we approached the warm lalitudes, a few, chiefly marines, who had never been in a hot climate before, complained of head-ach, and febrile fymptoms, which were removed by bleeding, and other evacuations. In March and April we were on our ftation at Port Royal. In May and June, when the periodical rains fet in, we were on a cruize, in confequence of which we did not fuffer from the rains or heat of thofe two months, and benefited by the refrefhing breezes met with at fea; and it has been frequently remarked, that mariners enjoy a greater degree of health, when at fea in the Weft Indies, than in port in any other part of the world. Commanding officers would therefore act judicioufly, in keeping fhips as little as poffible in harbour on that ftation. Commodore Gardner, then commanding his Majefty's fhips in that quarter of the globe, was in the habit of keeping the fquadron much at fea, which had a very falutary effect on the crew. During the whole of Auguft, September, and October, which are, what are called, the hurricane months, we went up the harbour, and anchored pretty high, towards Rock-Fort, to avoid the danger of thofe furious winds, which are fometimes attended with dreadful deftruction, both at fea and on fhore; this was experienced when I was at Jamaica, in October 1780; during thefe months deluges of rain fell, which obliged us to keep the awnings almoft conftantly fpread and floping; the fea-breezes were interrupted, and the atmofphere in general became exceffively clofe, fultry, and loaded with moifture, attended with thunder and lightning.

During this time, a languor and dejection of fpirits were felt by all on board; as the weather prevented any duty from being performed, and confcious of a predifpofition to difeafe, we judged it advifeable, to promote every kind of fport and diverfion. Dancing, that favourite amufement with feamen, was chearfully encouraged, as we fortunately had a fidler on board; a more ufeful man in a fhip, than a trumpeter, who I believe is generally allowed to his majefty's fhips, but for what purpofe, I have yet to learn.

In

In the beginning of November we ſailed to Port-Royal, without having a ſick man on board, or one left behind at the hoſpital; the three men ſent thither in September had returned to the ſhip. Such an extraordinary and uncommon ſtate of health, I cannot impute to any other circumſtance, but the ſalutary regulations abovementioned, which had been ſtrictly enforced: and above all, that the men were not allowed to go on ſhore, and thereby leſs expoſed to the exciting cauſes of diſeaſe.

While the ſhip lay anchored from the main land, as far as ſafety would permit, we enjoyed the benefit of the ſea-breeze, when it did not reach ſhips who were nearer the ſhore; for near theſe ſhips it frequently blows but faintly, and partially. In November and December, the weather becomes more ſettled; a freſh North wind blows in the day, from the continent of America, and in the night a ſtrong land-wind, carrying with it a conſiderable quantity of marſh-effluvia: at this time the greateſt ſickneſs generally prevails in the town of Kingſton. As the ſhips at Port-Royal are not entirely out of the reach of its influence, the beſt way to avoid it is, to put to ſea; the cool North winds un-impregnated with that noxious exhalation, will preſerve health, and ſpeedily recover convaleſcents; as we experienced in ſeveral caſes of remittent fever. The ſtate of the weather during the other two years, varied but little, except the hurricane months of 1789; we had not the uſual rains and ſqualls, which diſtinguiſh that ſeaſon: the ſea-breezes became more interrupted, and changeable; for the moſt part, indeed, a calm prevailed, and the heat was extreme: Fahrenheit's thermometer during the whole of September, was not under 90° in the ſhade; but in the beginning of October, a very conſiderable and ſudden change took place in the temperature of the air, which was followed by an epidemic catarrh in the ſquadron, and very prevalent among the troops and inhabitants on ſhore; very few eſcaped it; and as comparatively few perſons were attacked this year, with the endemic remittent fever, it was ſuppoſed by many, that the qualities of the air had undergone ſome change, which had produced this influenza, and prevented the prevalence of the fever. In my opinion, however, our exemption was owing to the want of the uſual quantity of rain, one of the neceſſary agents in producing marſh-miaſmata; Fahrenheit's thermometer, during the whole of our lying on the ſtation, ſeldom exceeded 82 or 83 on board, when not expoſed to the rays of the ſun.

*No.* 7, *Lancaſter-Court, Strand,* S. HENDERSON.

*Feb.* 20, 1799.

