# Erratum

**Published:** 1799-04

**Authors:** 


——93. l. 10, for 'were at the hospital,' read *died at the hospital.*

